# Anxiety and Mood Disturbance Are Prospectively Associated With Respiratory Infection Risk and the Mucosal Immune Response to Exercise

**DOI:** 10.1002/ejsc.70058

**Published:** 2025-09-30

**Authors:** Sophie E. Harrison, Jason P. Edwards, Ross Roberts, Neil P. Walsh

**Affiliations:** ^1^ Institute for Applied Human Physiology School of Sport Science Bangor University Bangor Wales UK; ^2^ Research Institute for Sport and Exercise Sciences Liverpool John Moores University Liverpool UK; ^3^ Institute for the Psychology of Elite Performance School of Sport Science Bangor University Bangor Wales UK

**Keywords:** anxiety, marathon running, mucosal immunity, SIgA, stress

## Abstract

We prospectively examined whether psychological factors influence (a) respiratory tract infection (RTI) risk and (b) the mucosal immune response to exercise. In Study 1, *n* = 406 adults (67% male) recorded RTI symptoms for two weeks before and after a marathon. In Study 2, under controlled laboratory conditions, *n* = 45 adults (51% male) completed 60 min of running at 65% V̇O_2peak_ (EX) and seated rest (CON) in randomised order. Anxiety, total mood disturbance (TMD) and perceived psychological stress were measured before exercise. Saliva collected pre‐ and post‐exercise was analysed for secretory immunoglobulin A (SIgA). Fifty runners suffered an RTI post‐marathon. Runners prospectively reporting high trait anxiety or TMD were more likely to suffer an RTI post‐marathon (OR [95% CI] = 1.06 [1.02–1.11] and 1.04 [1.01–1.07], respectively). Higher trait anxiety and TMD were associated with a greater reduction in saliva SIgA (*p* < 0.05). There was no association between mucosal immunity and RTI risk (OR [95% CI] = 1.00 [0.97–1.01]). In Study 2, despite no significant difference between EX and CON (*p* > 0.05), psychological factors were associated with the SIgA secretion rate response to exercise in men (trait anxiety, state anxiety, TMD, psychological stress: *r* = −0.55, −0.65, −0.61 and −0.66, respectively; *p* < 0.01). In conclusion, anxiety and mood disturbance were prospectively associated with infection risk after a marathon and the mucosal immune response to exercise. Athletes should optimise psychological well‐being to support immune health. Researchers should take account of psychological factors when examining the mucosal immune response to exercise.

## Highlights

- Psychological factors, including anxiety and mood disturbance, were prospectively associated with respiratory infection risk after a marathon and the mucosal immune response to exercise.
- Athletes should take steps to minimise exposure to high stress and anxiety and improve mood prior to competition to support immune health and infection resistance.
- Researchers should account for psychological stress and anxiety when examining the immune response to exercise.

## 1 Introduction

Marathon running has increased in popularity; in 2016, more than 1.8 million people worldwide ran a marathon (Doherty et al. 2020). Between 13% and 33% of runners report respiratory tract infection (RTI) after marathon or ultramarathon events (Nieman et al. 2006, 1990; Peters and Bateman 1983). RTI has a negative impact on the health and performance of athletes, limiting their ability to train and impairing performance (Palmer‐Green et al. 2013; Walsh, Gleeson, Pyne et al. 2011). Indeed, athletes who report fewer or shorter illnesses are more likely to compete at a higher level and be more successful (Hellard et al. 2015; Svendsen et al. 2016). Research in the field of sport and exercise immunology has focused on the role of physical stress on immunity and RTI susceptibility, showing greater risk of respiratory infection after marathon and ultramarathon events (Nieman et al. 2006, 1990; Peters and Bateman 1983). Other factors also influence immunity and RTI susceptibility, including poor nutrition, inadequate sleep and long‐haul travel (Ekblom et al. 2006; Svendsen et al. 2016; Walsh 2018; Wentz et al. 2018). Research in the more established field of psychoneuroimmunology has also shown that psychological stress and anxiety are associated with RTI susceptibility (Cohen et al. 1991, 1993; Dhabhar 2014; Smolderen et al. 2007). In athletes, a relationship between psychological factors and infection risk has been demonstrated, but as measures were collected retrospectively, reverse causation could not be ruled out (Drew et al. 2018). No studies have prospectively examined the association between psychological factors and RTI after prolonged exercise, which therefore requires investigation.

Mucosal surfaces are the site of infection or route of access for the majority of viruses and bacteria that cause RTI (Walsh, Gleeson, Shephard et al. 2011). Secretory immunoglobulin A (SIgA) is the most commonly assessed measure of mucosal immunity because it is the most abundant antimicrobial protein in saliva, with lower SIgA levels in saliva and tear relating to increased RTI risk (Hanstock et al. 2016; Hellard et al. 2015; Neville et al. 2008). Although some studies have shown transient decreases in SIgA after prolonged bouts of exercise and during periods of heavy training (Neville et al. 2008; Nieman et al. 2002; Peters and Bateman 1983; Walsh, Gleeson, Shephard et al. 2011), saliva SIgA responses to endurance exercise have often yielded discrepant findings, which have been attributed to differences in exercise modality, intensity and duration, as well as inconsistencies in saliva collection, handling, storage, analysis and reporting (Bishop and Gleeson 2009; Walsh, Gleeson, Shephard et al. 2011). The reported effects of both psychological stress and physical exertion on mucosal immunity are strikingly similar, whereby chronic psychological and prolonged physical stress decrease SIgA (Irshad et al. 2020; Phillips et al. 2006; Walsh, Gleeson, Shephard et al. 2011), and acute psychological stress and shorter exercise bouts have been shown to increase SIgA (Blannin et al. 1998; Clow and Hucklebridge 2001; Fan et al. 2009; Walsh, Gleeson, Shephard et al. 2011). Psychological stress and physical exertion are both well known to alter mucosal immunity through the hypothalamic‐pituitary‐adrenal (HPA) and sympathetic‐adreno‐medullary (SAM) axes (Kohut 2019; Perna et al. 1997). However, exercise immunologists have largely ignored the role of mental stress, despite physical exertion having a psychological component. Therefore, psychological factors likely play a role in modulating the immune response to exercise and, as such, may provide some explanation for previous discrepant findings when examining the mucosal immune response to exercise. Providing initial support, psychological stress and anxiety have been shown to influence immune activation after exercise (Edwards et al. 2018). As such, it seems likely that psychological factors play a role in the mucosal immune response to exercise, but such a relationship remains unknown and warrants investigation.

With this information in mind, here we present the findings from two studies examining whether psychological factors influence (a) RTI risk and (b) the mucosal immune response to exercise. In Study 1, we prospectively examined the association between psychological factors, and RTI risk and the mucosal immune response to a marathon race. In Study 2, we further examined the observed association between psychological factors and the mucosal immune response to exercise shown in Study 1 in a rigorously controlled laboratory setting.

## 2 Methods

Both studies received local university ethics committee approval and were conducted in accordance with the Declaration of Helsinki (2013) principles without pre‐registration.

### 2.1 Study 1

#### 2.1.1 Participants and Study Design

**FIGURE 1:**
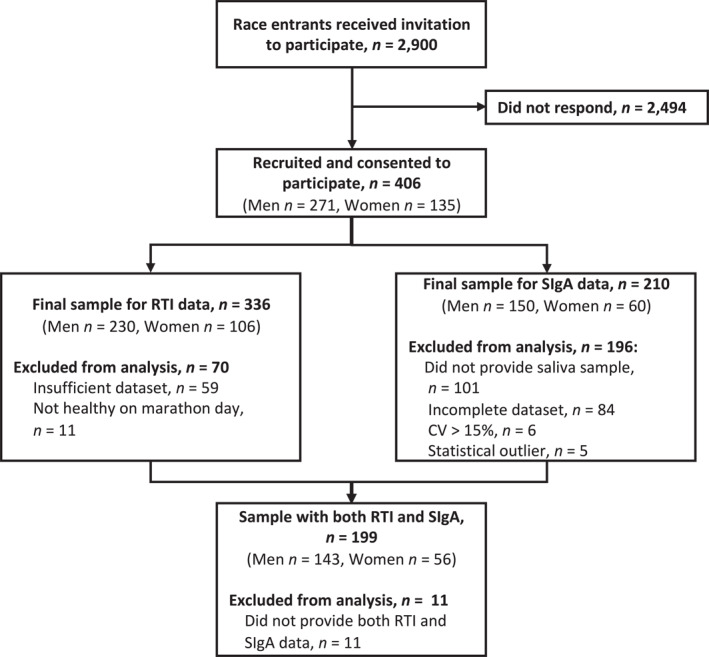
Flow chart of participant recruitment and those included in data analysis for Study 1 for respiratory tract infection (RTI) and mucosal immunity measured by salivary secretory immunoglobulin A (SIgA).

In this cohort observational study, a total of 2900 individuals taking part in the 2017 Snowdonia Marathon in Wales, UK, received an invitation to participate through email from race organisers and through recruitment at race registration the day before the marathon. Participants recruited at race registration were included for saliva collection and variables monitored at recruitment, on race day and during the two weeks after the race. Four hundred and six marathon runners (men: *n* = 271, age = 46 ± 9 years, height = 177 ± 12 cm, body mass = 78 ± 10 kg, BMI = 24 ± 3 kg·m^−2^; women: *n* = 135, age = 43 ± 8 years, height = 166 ± 6 cm, body mass = 62 ± 8 kg, BMI = 23 ± 3 kg·m^−2^) agreed to participate and provided informed consent (Figure 1).

#### 2.1.2 Marathon Race Setting and Experimental Procedures

The Snowdonia Marathon is an arduous mountainous race that is run on mixed terrain (road and trail) with a total ascent of 939 m. Demographic and trait anxiety data were collected upon recruitment prior to the marathon. RTI symptom data were reported twice per week during the two weeks before and after the marathon. Ten days prior to the marathon, total mood disturbance (TMD) was assessed. On the morning of the marathon, state anxiety and sleep duration from the previous night were assessed. The marathon started at 10:30 AM. Saliva samples were collected prior to and immediately after the marathon (9:16 AM ± 36 min and 3:11 PM ± 48 min, respectively).

#### 2.1.3 RTI Symptom Measurement (Study 1 Only)

RTI was monitored by self‐reported symptoms using the Jackson common cold questionnaire (Jackson et al. 1958). Participants were asked to rate eight symptoms (sneezing, headache, feeling generally unwell, runny nose, blocked nose, sore throat, cough, chilliness) on a four‐point Likert scale (not at all = 0, mild = 1, moderate = 2, severe = 3). Data were included when participants completed ≥ 80% of their Jackson questionnaires. An RTI was defined by a daily total symptom score of ≥ 6 for two or more consecutive days (Hanstock et al. 2016). To mitigate reverse causation, participants were excluded from statistical analysis if they reported a daily total symptom score of ≥ 6 on the day of the marathon (Figure 1).

#### 2.1.4 Sleep Duration Measurement (Study 1 Only)

Sleep duration was used as a covariate in Study 1 logistic regression analyses, as shorter sleep duration has previously been associated with increased RTI risk (Cohen et al. 2009; Prather et al. 2015). Sleep duration the night before the marathon was calculated based on self‐reported sleep and wake times.

### 2.2 Study 2

#### 2.2.1 Participants and Study Design

In a repeated measure, crossover design, 45 participants provided written informed consent to participate in this study (Table 1). Participants were recreationally active (3–9 h of aerobic exercise per week), nonsmokers and did not take prescription medication for the duration of the study or in the preceding month. Participants were healthy in the seven days preceding each experimental trial, that is, did not report any respiratory illness symptoms, and were asked to refrain from alcohol, caffeine, over‐the‐counter medication and heavy exercise during the 24 h before each experimental trial. Females were either naturally menstruating (23–32‐day menstrual cycle; *n* = 10; Cole et al. 2009) or using oral contraceptives (OC; *n* = 4) or a contraceptive implant (*n* = 5). OC users reported using Rigevidon (*n* = 2), Loette (*n* = 1) and Yaz (*n* = 1). To account for menstrual phase influencing mucosal immunity and responses to psychological measures, experimental trials were conducted on the same day of two separate cycles for naturally menstruating females and contraceptive implant users. Cycle day was determined by self‐reported calendar counting only. Oral contraceptive users completed both trials during active pill days (Days 9–28), within the same cycle or across two separate cycles.

**TABLE 1:** Demographic and psychological measures for all (men and women combined) and men and women separately in Study 2.

	All (*n* = 45)		Men (*n* = 26)		Women (*n* = 19)	
Age (years)	22 ± 3		21 ± 2		23 ± 4^a^	
Height (cm)	174 ± 9		180 ± 7		166 ± 6^a^	
Body mass (kg)	72 ± 10		76 ± 9		67 ± 10^a^	
BMI (kg·m^−2^)	24 ± 3		24 ± 3		24 ± 3	
Trait anxiety (AU)	38 ± 10		35 ± 8		43 ± 11^a^	

	Exercise	Seated rest	Exercise	Seated rest	Exercise	Seated rest
Total mood disturbance (AU)	103 ± 12	102 ± 11	102 ± 12^b^	99 ± 7	105 ± 13	107 ± 15^a^
State anxiety (AU)	34 ± 11	30 ± 9	32 ± 10^b^	28 ± 7	37 ± 12^a^	33 ± 11^a^

*Note:* Trait and state anxiety scores range from 20 to 80 AU, and total mood disturbance scores range from 84 to 180 AU.

^a^ *P* < 0.05 versus men.

^b^ *P* < 0.05 versus seated rest in men.

#### 2.2.2 Preliminary Measures and Familiarisation

Anthropometric measures were recorded on arrival at the laboratory before V̇O_2peak_ was estimated by means of a ramped exercise test on a treadmill (HP Cosmos Mercury 4.0; Nussdorf‐Traunstein, Germany). After 3 min of walking at 5 km·h^−1^ with an incline of 1%, speed increased at a rate of 1 km·h·min^−1^ to a maximum of 18 km·h^−1^, after which the incline increased at a rate of 1%·min^−1^ until volitional exhaustion. Pulmonary gas exchange was measured breath‐by‐breath for the duration of the test (Cortex MetaLyzer 3B, Biophysik, Leipzig, Germany), and V̇O_2peak_ was determined from the highest 30 s average, as described (Diment et al. 2015). The running speed that elicited 65% V̇O_2peak_ was then determined and verified using the interpolation of the running speed–V̇O_2_ relationship. All participants were familiarised with their running speed that elicited 65% V̇O_2peak_ for 10 min and saliva collection. Trait anxiety was also assessed during this visit.

#### 2.2.3 Experimental Procedures

In a randomised order, participants completed either 60 min of continuous treadmill running at 65% V̇O_2peak_ (EX) or remained seated for 60 min while watching a neutral video about trains (CON). Experimental trials were conducted in controlled conditions (20 ± 1°C, 43 ± 6% relative humidity, 764 ± 27 mmhg, fan speed 0.2 m·s^−1^ for EX) at the same time of day, with each trial separated by ≥ 48 h. Participants arrived at the laboratory fasted, and nude body mass was recorded before being provided with a standardised meal (2.2 MJ) and 5 mL·kg^−1^ nude body mass of water. Urine specific gravity was assessed before the exercise bout and seated rest to ensure participants were euhydrated (< 1.025 urine specific gravity) (Armstrong et al. 2016). During the exercise bout, 3 mL·kg·h^−1^ water was provided at 15, 30 and 45 min to offset fluid losses through sweating. During CON, participants were provided with the equivalent of 35 mL·kg·d^−1^ at 15, 30 and 45 min of the seated rest period (Hanstock et al. 2016). Perceived psychological stress, state anxiety and TMD were assessed prior to the exercise bout and seated rest, with saliva samples collected immediately pre‐, post‐ and 30 min post‐exercise and seated rest. Heart rate (HR) was monitored continuously throughout the 60‐min exercise and seated rest periods, with Borg’s rating of perceived exertion (RPE) recorded at 15, 30, 45 and 60 min of the exercise bout.

### 2.3 Psychological Measures

#### 2.3.1 State and Trait Anxiety

State and trait anxiety were assessed using the State‐Trait Anxiety Inventory (STAI), one of the most widely used measures to assess anxiety (Spielberger 1983). State anxiety (STAI‐S) is characterised by feelings of tension, apprehension, nervousness and worry in the moment, whereas trait anxiety (STAI‐T) reflects a relatively stable tendency to perceive stressful situations as threatening (Spielberger 1983). The STAI consists of 20 state items and 20 trait items, with responses being measured on a four‐point Likert scale, ranging from 1 ‘not at all’ to 4 ‘very much so’ for state anxiety and from 1 ‘almost never’ to 4 ‘almost always’ for trait anxiety. Scores ranged from 20 to 80 AU for each of the scales, with higher scores reflecting greater anxiety (composite reliability: STAI‐T Study 1 = 0.92, STAI‐T Study 2 = 0.92, STAI‐S Study 1 = 0.92, STAI‐S Study 2 = 0.94).

#### 2.3.2 Total Mood Disturbance

TMD in the past week was assessed using the Brunel Mood Scale (BRUMS; Terry et al. 2003). The BRUMS is a 24‐item measure that consists of five negative subscales (anger, confusion, depression, fatigue, tension) and one positive subscale (vigour), with responses measured on a five‐point Likert scale from 0 ‘not at all’ to 4 ‘extremely’. TMD was calculated by summing the scores for anger, confusion, depression, fatigue and tension and subtracting the score for vigour and then adding 100 to ensure all scores were greater than zero for ease of interpretation (Lastella et al. 2015), resulting in scores ranging from 84 to 180 AU, with higher scores reflecting greater TMD (composite reliability: Study 1: anger = 0.81, confusion = 0.87, depression = 0.92, fatigue = 0.90, tension = 0.88, vigour = 0.86; Study 2: anger = 0.95, confusion = 0.84, depression = 0.81, fatigue = 0.84, tension = 0.92, vigour = 0.90).

#### 2.3.3 Perceived Psychological Stress (Study 2 Only)

Perceived psychological stress in the past month was assessed using the perceived stress scale (PSS); the PSS is the most widely used psychological instrument for measuring the perception of stress and measures the degree to which life situations are considered stressful by the individual (Cohen et al. 1983). The PSS is a 10‐item inventory, with responses measured on a five‐point Likert scale from 0 ‘never’ to 4 ‘very often’ with a range of scores from 0 to 40 AU, with higher scores reflecting greater perceived psychological stress (Study 2 composite reliability = 0.87).

### 2.4 Saliva Collection and Analysis

In Study 1, saliva samples were collected for 2 min using a preweighed Versi‐SAL swab (Oasis Diagnostics, Vancouver, WA, USA), whereby the participant placed the swab under the tongue, with their mouth kept closed for the duration of sample collection (Fortes et al. 2012). Saliva samples were collected by Versi‐SAL swab in Study 1 for practical reasons to allow a large number of samples to be collected within a short window in an environment where chairs were not readily available to perform a passive drool sample. Samples were temporarily stored at 4°C for transportation from the marathon location to the laboratory before being weighed. Pilot data (Hanstock et al. 2016 unpublished observation) within our laboratory demonstrated SIgA concentrations remained stable at 4°C for at least 3 h (journey duration ∼ 20 min). In Study 2, saliva samples were collected for 5 min by the participant leaning forward and passively drooling into a preweighed universal tube with minimal orofacial movements, as described (Oliver et al. 2007). Samples were weighed after collection, with saliva collected for a further 2 min (up to a maximum of 9 min) if less than 200 μL volume was obtained after 5 min. In both studies, samples were centrifuged at 1500 g and 4°C for 10 min, aliquoted and stored at −80°C until analysis. Samples were analysed for SIgA using enzyme‐linked immunosorbent assay (ELISA, Salimetrics, Pennsylvania, USA). Mean intra‐assay CVs for sample duplicates were 2.9% and 3.3% for Study 1 and Study 2, respectively. Saliva flow rate (FR) was calculated by dividing saliva volume by total collection time. Assuming the density to be 1.00 g·ml^−1^ for saliva, the SIgA secretion rate (SR) was calculated by multiplying saliva FR by saliva SIgA concentration (Oliver et al. 2007). Pilot data within our laboratory demonstrated similar SIgA concentrations and SRs using the ‘passive drool’ and Versi‐SAL swab collection methods.

### 2.5 Statistical Analysis

Statistical analyses were performed using SPSS 29.0 (IBM, Chicago, IL). Data are presented as mean ± SD unless otherwise stated, with statistical significance accepted as *p* < 0.05. Mucosal immune data (saliva FR, SIgA concentration and SIgA SR) were checked for normal distribution and, in cases where the assumption of normality was violated, were square‐root transformed, with outliers more than three times the interquartile range removed prior to analysis (Study 1: *n* = 5; Study 2: *n* = 3; Field 2013).

In Study 1, paired samples *t*‐tests were performed to compare pre‐ and post‐marathon saliva FR, SIgA concentration and SIgA SR. Hierarchical logistic regression was used to examine the relationship between psychological factors and RTI during the two weeks after the marathon. Hierarchical linear regression was used to assess the relationship between trait anxiety, state anxiety and TMD, and the mucosal immune response to exercise (pre‐post exercise change in saliva FR, SIgA concentration and SIgA SR). Logistic regression was used to examine the relationship between the mucosal immune response to the marathon and RTI during the two weeks after the marathon. For all logistic and linear regression analyses, adjustments for covariates previously shown to influence RTI or mucosal immunity were made as follows: sex, age, BMI, marathon race time and sleep duration the night before the marathon; when RTI was an independent variable, adjustment was made for RTI incidence during the two weeks before the marathon (Ekblom et al. 2006). We then hypothesised that there may be an interactive effect between trait and state anxiety, whereby those reporting both high trait and high state anxiety might be at greater risk of RTI. For these analyses, we based our analytical strategy on guidelines for using interaction and main effects only models in regression (Gaudreau 2012). For each hypothesis, we first performed moderated logistic binary regression to establish whether the interaction between RTI and each of the moderator variables (trait anxiety and state anxiety) was statistically significant. In the absence of a significant interaction, following Gaudreau’s suggestions (Gaudreau 2012; Gaudreau et al. 2022), we removed the interaction term and then ran binary logistic regression models that included only the two main effects (RTI and trait or state anxiety). We then plotted predicted odds ratio values for each combination of the variables. As Gaudreau notes (Gaudreau 2012; Gaudreau et al. 2022), such an approach is appropriate to test hypotheses of this nature because it allows predicted values of varying combinations of the independent variables (low–low, low–high, high–low, high–high; −1 SD and +1 SD reflect ‘low’ and ‘high’ values of the constructs, respectively) to be calculated, therefore providing researchers with good evidence as to whether their hypotheses can be supported or not.

In Study 2, independent samples *t*‐tests were used to compare demographic and psychological characteristics between men and women, with repeated measures ANOVA used to examine differences in saliva FR, SIgA concentration and SIgA SR between EX and CON (group × time). Pearson’s correlations were used to examine the relationship between trait anxiety, perceived psychological stress, state anxiety and TMD, and the mucosal immune response to exercise (pre‐post exercise change in saliva FR, SIgA concentration and SIgA SR) in all participants and men and women separately.

## 3 Results

### 3.1 Study 1

The Snowdonia Marathon is an arduous mountain race that is run on mixed terrain with a total ascent of 939 m. Reflecting the arduous nature of this race, the winning times in the Snowdonia Marathon were over 2.5 h for men and almost 3 h for women (men: 2 h 26 min, women: 2 h 57 min). In Study 1, the average completion time was 4 h 26 min ± 46 min for men and 4 h 55 min ± 49 min for women. State anxiety levels on the morning of the race were high (40 ± 11 AU; Julian 2011). Women reported higher trait anxiety, TMD and state anxiety compared with men (trait anxiety: 39 ± 10 vs. 37 ± 9 AU, *p* = 0.022; TMD: 111 ± 14 vs. 106 ± 13 AU, *p* = 0.003; state anxiety: 43 ± 12 vs. 38 ± 10 AU, *p* < 0.001). Compared with pre‐marathon, saliva FR and SIgA SR decreased and SIgA concentration increased post‐marathon (Supporting Information S1: Table S1; all *p* < 0.001). During the two weeks after the marathon, 15% of runners suffered an RTI (*n* = 50).

#### 3.1.1 The Association Between Psychological Factors and RTI After a Marathon Race

**FIGURE 2:**
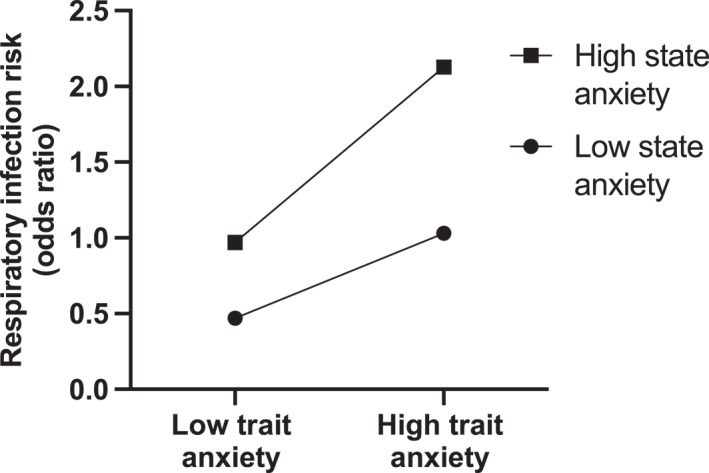
Interaction between trait anxiety and state anxiety on respiratory infection risk during the two weeks after the marathon (Study 1).

Trait anxiety and TMD prior to the marathon predicted increased RTI susceptibility during the two weeks after the marathon, after adjustment for covariates (sex, age, BMI, race duration, sleep duration and pre‐marathon RTI; Table 2; *p* = 0.009 and 0.012, respectively). There was no relationship between state anxiety and RTI after the marathon (Table 2; *p* > 0.05). Moderated logistic regression showed no interaction between trait anxiety and state anxiety on RTI susceptibility (*p* > 0.05). Trait anxiety, but not state anxiety, was associated with an increased risk of RTI (Table 2; *p* = 0.009 and *p* > 0.05, respectively). However, runners reporting high trait anxiety had greater odds of suffering an RTI after the marathon if they reported high state anxiety compared to runners reporting low state anxiety (Figure 2).

**TABLE 2:** The association between trait anxiety, total mood disturbance and state anxiety, and respiratory tract infection (RTI) during the two weeks after the marathon race (Study 1) in unadjusted and adjusted hierarchical logistic regression.

RTI post‐marathon	OR (95% CI)	
Predictor	Unadjusted	Adjusted
Trait anxiety	1.04* (1.00–1.07)	1.06* (1.02–1.11)
Total mood disturbance	1.04* (1.01–1.06)	1.04* (1.01–1.07)
State anxiety	1.02 (0.99–1.04)	1.03 (0.99–1.07)

*Note:* The adjusted model includes sex, age, body mass index, race time, sleep duration and RTI during the two weeks before the marathon in prior steps.

^*^ *p* < 0.05.

#### 3.1.2 The Association Between Psychological Factors and the Mucosal Immune Response to a Marathon Race

Higher trait anxiety and TMD were associated with a greater reduction in saliva SIgA SR in response to the marathon (Table 3; trait anxiety: *p* = 0.010, TMD: *p* = 0.011). No associations were observed between state anxiety and SIgA SR (Table 3; *p* > 0.05). No associations were observed between trait anxiety, TMD and state anxiety, and saliva FR or SIgA concentration (Table 3; *p* > 0.05).

**TABLE 3:** The association between trait anxiety, total mood disturbance and state anxiety, and the mucosal immune response (saliva flow rate [FR], SIgA concentration and SIgA secretion rate [SR]) to a marathon (change pre‐post marathon), after accounting for covariates (sex, age, body mass index, race time and sleep duration; Study 1).

Change pre‐post marathon	Trait anxiety				Total mood disturbance				State anxiety			
	B	SE	R2	∆ R2	B	SE	R2	∆ R2	B	SE	R2	∆ R2
Saliva FR	−0.053	0.028	0.083	0.019	−0.023	0.023	0.074	0.006	−0.022	0.027	0.081	0.004
Saliva SIgA concentration	0.004	0.020	0.039	0.000	−0.016	0.017	0.034	0.006	−0.013	0.019	0.046	0.003
Saliva SIgA SR	−0.008	0.003	0.144	0.033*	−0.007	0.003	0.161	0.039*	−0.005	0.003	0.133	0.016

^*^ *p* < 0.05.

#### 3.1.3 The Association Between Mucosal Immunity and RTI

There was no association between saliva flow rate, SIgA concentration or SIgA SR change pre‐post marathon and RTI during the two weeks after the marathon (saliva flow rate: OR [95% CI] 1.00 [1.00–1.00], SIgA concentration: OR [95% CI] 1.00 [1.00–1.00], SIgA SR: OR [95% CI] 1.00 [0.97–1.01]; *p* > 0.05).

### 3.2 Study 2

Perceived psychological stress and TMD prior to exercise were similar between EX and CON, with higher anticipatory levels of state anxiety before EX (Table 1). Perceived psychological stress and state anxiety levels were low to moderate, comparable with a young adult population (Cohen and Janicki‐Deverts 2012; Spielberger 1983). As in Study 1, women reported higher levels of trait anxiety, perceived psychological stress and state anxiety, and greater TMD than men (Table 1, *p* < 0.05).

As expected, HR was higher during EX compared to CON (EX: 159 ± 12; CON: 69 ± 16 bpm; *p* < 0.001) and rose steadily during the exercise bout (15 min: 154 ± 13, 30 min: 161 ± 12, 45 min: 162 ± 13, 60 min: 161 ± 23 bpm). Participants reported an end‐exercise RPE of 13 ± 3 (‘somewhat hard’). The exercise bout did not lead to any changes in saliva FR, concentration or SIgA SR compared to CON (Supporting Information S1: Table S2; *p* > 0.05). Saliva FR and SIgA SR were lower overall during EX versus CON in men and women combined and in men only, but not in women only (Supporting Information S1: Table S2).

#### 3.2.1 The Relationship Between Perceived Psychological Stress, Anxiety and TMD, and the Mucosal Immune Response to 60 min of Moderate‐Intensity Exercise

**FIGURE 3:**
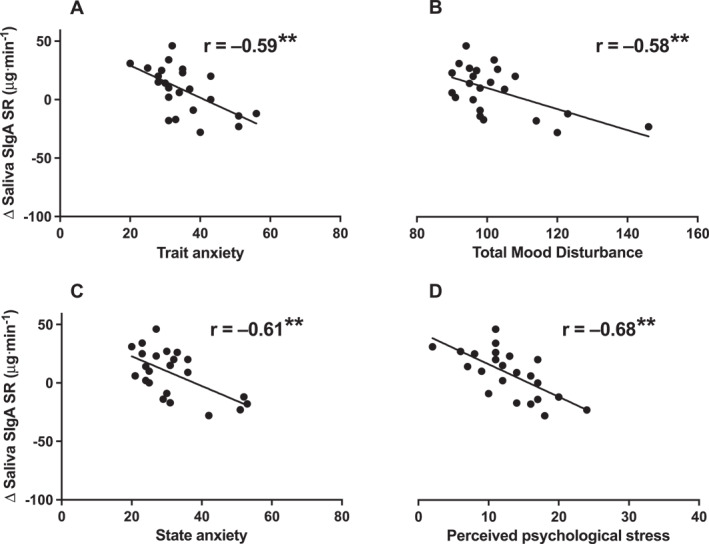
The relationship between prospectively measured trait anxiety (A), total mood disturbance (B), state anxiety (C), perceived psychological stress (D) and the mucosal immune response to 60 min of moderate‐intensity exercise in men (Study 2). **p < 0.01; The mucosal immune response to exercise represents the pre‐post change to exercise. SIgA = secretory immunoglobulin A, SR = secretion rate.

Perceived psychological stress, trait anxiety, state anxiety and TMD were not associated with the mucosal immune response to exercise when examining both men and women combined (*p* > 0.05). However, in men, we showed negative associations between trait anxiety, TMD, state anxiety, perceived psychological stress and the saliva SIgA SR response to exercise (Figure 3; trait anxiety: *p* = 0.003; TMD, state anxiety and perceived psychological stress: *p* < 0.001). Trait anxiety, perceived psychological stress, TMD and state anxiety were also negatively associated with saliva FR responses to exercise (Supporting Information S1: Figures S1–S4; trait anxiety and perceived psychological stress: *p* < 0.001; TMD *p* = 0.007; state anxiety *p* = 0.005). Participants reporting higher anxiety or stress had a decrease in saliva SIgA SR, and participants reporting lower anxiety or stress had an increase in SIgA SR after exercise. In men, we did not see an influence of psychological factors on the SIgA concentration response to exercise (Supporting Information S1: Figures S1 and S2; *p* > 0.05).

In women, state anxiety and TMD were positively correlated with the SIgA SR response to exercise (Supporting Information S1: Figures S3 and S4; *p* = 0.018 and 0.017), and TMD was also positively correlated with the SIgA concentration response to exercise (Supporting Information S1: Figure S4; *p* = 0.027). Perceived psychological stress and state anxiety were related to the saliva FR response to exercise in women (Supporting Information S1: Figures S1 and S3; both *p* = 0.043). However, perceived psychological stress and trait anxiety were not related to the mucosal immune response to exercise in women (Supporting Information S1: Figures S1 and S3; both *p* > 0.05).

## 4 Discussion

In support of our hypothesis, we show for the first time that psychological factors, including anxiety and mood disturbance, are prospectively associated with increased RTI risk after a marathon and the mucosal immune response to both laboratory‐ and field‐based exercise. In Study 1, we found a relationship between psychological factors (trait anxiety and mood disturbance) and respiratory infection in an athletic population. In Studies 1 and 2, we showed a negative association between psychological factors (trait anxiety, state anxiety, mood disturbance and psychological stress) and the mucosal immune response to exercise in both field‐ and rigorously controlled laboratory‐based studies. However, we did not find an association between mucosal immunity and RTI risk. Our novel findings suggest that, where possible, athletes should take steps to improve their psychological well‐being to support immune health and infection resistance. Taken together with previous work (Edwards et al. 2018), these findings support the recommendation that researchers should account for psychological stress, anxiety and mood when examining the immune response to exercise and may help to shed light on previous contradictory findings for the mucosal immune response to exercise within the literature.

In Study 1, trait anxiety and TMD were prospectively associated with increased respiratory infection risk during the two weeks after an arduous mountainous marathon race. These findings can be considered robust, as the associations remained after accounting for known respiratory infection risk factors (sex, age, BMI, race duration, sleep duration and prior respiratory infection). Further, in contrast to previous work (Drew et al. 2018), our findings are strengthened, as we collected prospective measures of psychological factors. Our findings align with and build on previous research in nonathletic populations showing that higher psychological stress is associated with increased respiratory infection risk (Cohen et al. 1991, 1993). Of additional interest, we found that runners reporting high trait and high state anxiety were at greater risk of respiratory infection than those reporting a combination of high trait and low state anxiety (Figure 2). These findings highlight that interventions to reduce anxiety and improve mood prior to exercise may support immune health and infection resistance, but further research should examine interventions in randomised control trials.

Building on previous work examining immune activation (Edwards et al. 2018), we found that anxiety, mood and psychological stress played a role in the mucosal immune response to exercise in both field‐ and laboratory‐based studies. In Study 1, higher trait anxiety and TMD were associated with a greater reduction in mucosal immunity after a marathon race. In agreement, in a rigorously controlled laboratory study, we showed a negative association between trait anxiety, TMD, state anxiety and perceived psychological stress, and the mucosal immune response to exercise in men. When examining exercise immunology research, only a handful of studies have accounted for psychological factors (Edwards et al. 2018; Huang et al. 2010; Moreira et al. 2011; K. Rehm et al. 2016; K. E. Rehm et al. 2013). Our findings highlight that psychological factors may play a role in explaining the previous discrepant findings for the mucosal immune response to exercise (Bishop and Gleeson 2009; Walsh, Gleeson, Shephard et al. 2011). The similar influence of psychological and physical stress on mucosal immunity is likely explained by shared effector limbs for the body’s response to various stressors, namely, the HPA and SAM axes (Walsh 2018). Indeed, there is likely a significant psychological stress component in prolonged heavy physical exertion, and as such, the previously observed immune responses to exercise are likely a result of the combined influence of both physical and psychological stressors.

We observed a relationship between psychological factors and respiratory infection, and separately, psychological factors and the mucosal immune response to a marathon. However, there was no association between the mucosal immune response to exercise and respiratory infection incidence during the two weeks after the marathon, unlike previous literature, which has shown a relationship between reduced salivary and tear SIgA and respiratory infection risk (Hanstock et al. 2016; Hellard et al. 2015; Neville et al. 2008). One reason for this discrepancy may be that we did not establish a baseline, as in previous studies (Hanstock et al. 2016; Hellard et al. 2015; Neville et al. 2008). Due to the field‐based setting of Study 1, we were unable to collect saliva samples prior to race day, which means that samples may have been influenced by anticipatory stress. Secondly, we could not account for pathogen exposure or participant hygiene behaviours. Further, respiratory infection was measured across a 2‐week period after the marathon, whereas the mucosal immune changes after the marathon were likely transient and short‐lasting and therefore may not relate well to infection risk across a longer time period. However, it should also be considered that reduced mucosal immunity may not be the sole or predominant mechanism for increased RTI risk in those reporting higher psychological anxiety or stress or greater mood disturbance. Anxiety and stress influence immunity through the HPA and SAM axes, which act upon several immune parameters (Kohut 2019; Perna et al. 1997). Saliva SIgA as an isolated measure within and between people may not predict RTI. Future studies should explore SIgA in other biofluids or the use of a more comprehensive oral immunity panel, as well as consider the HPA and SAM axes and the influence of other immune parameters (Campbell and Turner 2018).

Although this study was not designed to examine the role of sex, we found a consistent relationship between psychological factors and mucosal immunity in men but did not replicate this relationship in women in Study 2. In women, these findings were mixed, but it is currently difficult to explain such disparate findings. The current findings show that male and female responses may be opposing, which may provide further explanation for previous discrepant findings for the mucosal immune response to acute bouts of exercise in studies including both male and female participants but not taking account of potential sex differences (Bishop and Gleeson 2009). Indeed, male‐female differences are observed for the immune response to exercise, sleep and mental stressors, although findings are often discrepant (Besedovsky et al. 2022; T. Gillum et al. 2014; T. L. Gillum et al. 2011; He et al. 2014; Rutherfurd‐Markwick et al. 2017; Spiegel et al. 2023; Willemsen et al. 2002). In contrast to Study 2, we observed effects in a mixed cohort in Study 1. However, the average age of participants was higher in Study 1 compared to Study 2 (43 vs. 23 years), and as such, participants in Study 1 may be perimenopausal, menopausal or postmenopausal and may therefore have lower levels of oestrogen and progesterone, which may suggest a potential role for the hormonal milieu of oestrogen and progesterone; women have different cellular immune responses to exercise during different phases of their menstrual cycle (Hicks et al. 2023). As we did not determine cycle phase or measure oestrogen or progesterone, we cannot confirm the role of these hormones or specific menstrual cycle phases. Future research should examine sex differences and female‐specific immune responses to exercise.

We acknowledge that the findings of this study are restricted to association, and therefore, causation cannot be determined. Notwithstanding, confidence in our findings is increased because the relationships between psychological factors and both salivary SIgA and respiratory infection remain after accounting for respiratory infection risk factors (sex, age, BMI, race duration, sleep duration, prior infection). Further, steps were taken to mitigate reverse causation by excluding participants reporting RTI symptoms on the day of the marathon and prospectively measuring psychological factors. We also acknowledge that although our population is athletic, participants in this study were not elite athletes, and the average age of participants was relatively high in Study 1. As such, future research should seek to determine whether these findings translate to elite athletes. Finally, we acknowledge that respiratory symptoms were self‐reported, and we did not confirm infectious aetiology with pathology. It is possible that participants reporting poorer psychological well‐being have a tendency to report more physical symptoms (Suls and Howren 2012). However, previous research using the same common cold criteria showed that more than 80% of common colds were pathology confirmed, and research in 200 Swedish students showed that more than 92% of those reporting symptoms were pathology confirmed in autumn, the same season that our data were collected (Hanstock et al. 2016; Mäkelä et al. 1998).

## 5 Conclusion

In conclusion, psychological factors, including anxiety and mood disturbance, were prospectively associated with infection risk after a marathon and the mucosal immune response to laboratory‐ and field‐based exercise. Where possible, athletes should take steps to minimise exposure to high stress and anxiety and improve mood prior to competition to support immune health and infection resistance. Further, the findings of Studies 1 and 2, which incorporate both field‐ and laboratory‐based exercise, shed light on previously contradictory findings for the mucosal immune response to exercise. Taken together with previous work (Edwards et al. 2018), these findings support the recommendation that researchers should account for psychological stress and anxiety when examining the immune response to exercise.

## Conflicts of Interest

The authors declare no conflicts of interest.

## Supporting information


Supporting Information S1

## Data Availability

The results of Study 1 and Study 2 are presented clearly, honestly and without falsification or inappropriate data manipulation.
