# Supplementary material for: Anxiety and Mood Disturbance Are Prospectively Associated With Respiratory Infection Risk and the Mucosal Immune Response to Exercise
Source: Eur J Sport Sci. 2025 Sep 30;25(10):e70058. doi: 10.1002/ejsc.70058 (PMC12480926; doi:10.1002/ejsc.70058)
Supplement: Supplementary file 1 — Supporting Information S1 [file EJSC-25-e70058-s001.docx]

**Supplementary Figures**

**Supplemental Figure 1.** The relationship between perceived psychological stress and the mucosal immune response to 60 min moderate-intensity exercise (Study 2). Panels A & D present saliva flow rate, panels B & E present SIgA concentration and panels C & F present SIgA secretion rate response to exercise in men and women, respectively. **P* < 0.05; ***P* < 0.01; FR = flow rate; SIgA = secretory immunoglobulin A; SR = secretion rate. The mucosal immune response to exercise represents the pre-post change to exercise.

**Supplemental Figure 2.** The relationship between trait anxiety and the mucosal immune response to 60 min moderate-intensity exercise (Study 2). Panels A & D present saliva flow rate, panels B & E present SIgA concentration and panels C & F present SIgA secretion rate response to exercise in men and women, respectively. **P* < 0.05; ***P* < 0.01; FR = flow rate; SIgA = secretory immunoglobulin A; SR = secretion rate. The mucosal immune response to exercise represents the pre-post change to exercise.

**Supplemental Figure 3.** The relationship between state anxiety and the mucosal immune response to 60 min moderate-intensity exercise (Study 2). Panels A & D present saliva flow rate, panels B & E present SIgA concentration and panels C & F present SIgA secretion rate response to exercise in men and women, respectively. **P* < 0.05; ***P* < 0.01; FR = flow rate; SIgA = secretory immunoglobulin A; SR = secretion rate. The mucosal immune response to exercise represents the pre-post change to exercise.

**Supplemental Figure 4.** The relationship between total mood disturbance and the mucosal immune response to 60 min moderate-intensity exercise (Study 2). Panels A & D present saliva flow rate, panels B & E present SIgA concentration and panels C & F present SIgA secretion rate response to exercise in men and women, respectively. **P* < 0.05; ***P* < 0.01; FR = flow rate; SIgA = secretory immunoglobulin A; SR = secretion rate. The mucosal immune response to exercise represents the pre-post change to exercise.

**Supplementary tables**

**Supplemental Table 1.** Saliva flow rate (FR), SIgA concentration and SIgA secretion rate (SR) pre and post a marathon race in all participants, men and women (Study 1).

|  |  | All | Men | Women |
| --- | --- | --- | --- | --- |
| Saliva FR (µl·min^−1^)^a,b,c^ | Pre | 266 ± 208 | 281 ± 226 | 227 ± 148 |
|  | Post | 91 ± 65 | 85 ± 59 | 105 ± 76 |
| Saliva SIgA concentration (µg·ml^−1^) ^a,b,c^ | Pre | 165 ± 101 | 175 ± 106 | 139 ± 83 |
|  | Post | 247 ± 156 | 247 ± 151 | 250 ± 171 |
| Saliva SIgA SR (µg·min^−1^) ^a,b^ | Pre | 33 ± 24 | 36 ± 25 | 26 ± 19 |
|  | Post | 19 ± 15 | 19 ± 14 | 21 ± 15 |

a, different to pre in all, *P* < 0.001; b, different to pre in men, *P* < 0.001; c, different to pre in women, *P* < 0.001

**Supplemental Table 2.** Saliva flow rate (FR), SIgA concentration and SIgA secretion rate (SR) pre, post and 30 min post 60 min of treadmill running at 65% V̇O_2peak_ (exercise) and seated rest in all participants, and men and women separately (Study 2).

|  |  | All | | Men | | | Women | |
| --- | --- | --- | --- | --- | --- | --- | --- | --- |
|  |  | Seated Rest | Exercise | | Seated Rest | Exercise | Seated Rest | Exercise |
| Saliva FR (µl·min^−1^)^a,c,f,g,h^ | Pre | 207 ± 174 | 159 ± 139 | | 227 ± 188 | 171 ± 134 | 181 ± 154 | 144 ± 148 |
|  | Post | 234 ± 198 | 171 ± 143 | | 270 ± 205 | 210 ± 168 | 187 ± 185 | 122 ± 83 |
|  | +30 min | 225 ± 174 | 211 ± 169 | | 261 ± 184 | 223 ± 174 | 178 ± 153 | 196 ± 166 |
| Saliva SIgA concentration (µg·ml^−1^)^b,d,e^ | Pre | 204 ± 111 | 208 ± 102 | | 211 ± 116 | 212 ± 101 | 194 ± 107 | 203 ± 106 |
|  | Post | 232 ± 116 | 228 ± 120 | | 229 ± 113 | 239 ± 117 | 236 ± 124 | 212 ± 127 |
|  | +30 min | 192 ± 95 | 206 ± 100 | | 201 ± 97 | 226 ± 92 | 178 ± 93 | 176 ± 107 |
| Saliva SIgA SR (µg·min^−1^)^a,b,c,f,g,h^ | Pre | 40 ± 33 | 30 ± 26 | | 45 ± 36 | 31 ± 24 | 34 ± 27 | 29 ± 30 |
|  | Post | 46 ± 32 | 34 ± 25 | | 50 ± 31 | 40 ± 29 | 39 ± 33 | 25 ± 14 |
|  | +30 min | 40 ± 29 | 42 ± 32 | | 46 ± 31 | 46 ± 34 | 32 ± 26 | 35 ± 30 |

a, main effect of trial for all, *P* < 0.05; b, main effect of time for all (pre vs post), *P* < 0.05; c, main effect of time for all (pre vs 30 min post), *P* < 0.05; d, main effect of time for women (pre vs post), *P* < 0.05; e, main effect of time for women (pre vs 30 min post), *P* < 0.05; f, main effect of trial for men, *P* < 0.05; g, main effect of time for men (pre vs post), *P* < 0.05; h, main effect of time for men (pre vs 30 min post), *P* < 0.05
